# The relative age effect in selection of ab initio pilots–Revisiting Gladwell's famous claims

**DOI:** 10.1016/j.heliyon.2024.e37456

**Published:** 2024-09-12

**Authors:** Emil Lager, Kimmo Sorjonen, Marika Melin

**Affiliations:** Department of Clinical Neuroscience, Karolinska Institutet, 171 77, Stockholm, Sweden

**Keywords:** Relative age effect, Pilot selection, Operational ability, Cognitive ability, Maturational hypothesis

## Abstract

A relative age effect (RAE) has been found in education and sports, with students born earlier in the school year attaining higher levels of success. This was brought into public consciousness through Malcom Gladwell's *Outliers: The Story of Success.* However, the effect has not been thoroughly examined in other contexts. Thus, with regards to the context dependency of the RAE, and the specificity of which abilities it affects, there are unknowns. The aim of the present study was to scrutinize the tests in use in commercial aviation and explore whether there is a bias based on birthdate in competitive selection procedures assessing operational and cognitive abilities, outside of traditional education. Potential suboptimal selection has implications for flight safety. Associations were studied between relative age and test scores (of cognitive ability, manual spatial ability, and multitasking ability), as well as acceptance for higher education in commercial aviation in Sweden, in 1113 ab initio candidates. Relative age neither had a significant correlation with any of the six performance scores, nor with an aggregated factor score (*r* = −0.009, *p* = 0.791), nor predicted flight-school admission (*b* = 0.036, *p* = 0.210). Chronological age was associated with manual spatial ability, yet neither correlated with aggregate score nor with admission. The results may be an indication of the RAE having faded in early adulthood, and/or that indirect exposure of education does not benefit candidates in operational tests in early adulthood. Thus, neither relative age nor chronological age seemed to bias the results. For the effects of RAE to persist– or occur in operational abilities– competition between candidates in selection and subsequent relevant exposure (e.g., training/schooling), may be needed. Overall cognitive ability is likely more predictive of success when variance in specific enhancing exposure is limited.

## 1 Introduction

The relative age effect (RAE) is the phenomenon that those born early in the calendar year/close to cut-off, have a significantly higher probability of attaining higher levels of physiological, and psychological abilities, compared to those born later. This effect was brought into public consciousness largely due to Malcom Gladwell's book *Outliers: The Story of Success* [1]. The RAE has been found in numerous contexts. It has been identified in adult ice-hockey players [2], youth ice-hockey players [3], in professional soccer [4], among youth chess players, both in terms of actual participation and probability of high-ranking [5], as well as in alpine skiers [6], to name a few.

The RAE has also been studied in education. Relatively younger kids are more likely to experience selection disadvantages compared to their relatively older peers [7]. The RAE has been found to contribute to differences in attainment between children irrespective of gender, stage of education [8,9], or subject, with the discrepancies being at their peak at 11–12 year of age [10]. RAE perseveres through the educational system for math, science, and physical education [7]. Positive effects on different educational outcomes, including test scores, have been found for children entering school at 7 instead of 6 years of age. Older students in that cohort had a 12 % higher probability of attending the highest secondary schooling track [11]. Relatively older students have been found to be more likely to be tracked into academic education [12]. It has been reported that children who enter kindergarten at a relatively older age perform better in subsequent school years [13]. However, there are findings pointing in another direction. While relatively younger children tend to underperform in the first years of schooling, they have been found to complete higher levels of education to the same extent as their relatively older peers [14].

Different explanations have been presented to explain the differences in attainment where they occur. The seasonality hypothesis argues that exposure to winter infections leads to defects [15]. Yet, that explanation seems restricted to neurologically impaired children [16]. The staggered entry point/maturational hypothesis argues that pupils who get less time and experience in primary education fall behind [17]. This hypothesis has been vindicated by findings during initial years of education [18]. It has been found that advantages accumulate, the so called Matthew Effect (see Merton [19]). This effect has been found in education [20]. As selection dates change, achievement advantages have been found to shift accordingly [21]. Yet, the maturational hypothesis has been found to hold better when standardized entry is controlled for, and within-year maturational variation is most evident in sport [22]. In puberty, the RAE has been found to persist and even be accentuated [23]. However, others argue that the RAE only yields near-term benefits that fade over time [24]. It has been suggested that part of the reason that RAE sometimes persists lies in differences in skill accumulation, softer skills, and preparation [25]. Yet, a study created in response to parents enrolling a child into kindergarten at a later stage, found no RAE in completion of a research doctorate [26]. However, achieving a doctorate is rare, looking at such a group may mean looking at a strong and privileged group where other factors simply matter more. The RAE may be of more interest for lower levels of attainment or more straight-forward career paths, such as aviation. Candidates may delay applying for flight school to try and maximize the likelihood of entry. This assumption may seem speculative, yet some types of higher education [in Sweden] can only be applied for once. In some contexts, a prior [weak] application may lower the probability of later entry. In sum, the RAE has been found in multiple contexts, yet the understanding of the context dependency of the effect and which specific abilities it affects remains largely uncharted.

In high-stakes fields such as aviation, it is crucial to investigate whether the selection methods and tests employed are reliable and not susceptible to bias at the time of selection. When admitted to professional educational programs, operational and cognitive selection tests are conducted, and these assessments may potentially be influenced by the RAE. The aim of the present study is to scrutinize the tests in use in commercial aviation and explore whether there is a bias based on birthdate and how it might impact outcomes and by extension flight safety.

Are pilots born earlier in the year more likely to perform better in aptitude tests and/or be selected for training programs? Understanding any role RAE may play has implications for educational policies and practices. Educators and institutions may need to be aware of potential biases in selection processes and take measures to mitigate the impact of age-related differences on student outcomes. Despite the fertile ground to explore these questions, because of the richness of the test data, RAE has not been explored in aviation or any other “operative” profession previously, to our knowledge. In ab initio commercial pilot selection, the RAE can be studied from a unique perspective since the tests in use were designed to capture abilities that are not meant to depend on learned skills from the educational system. Perceptual tests and multitasking tests weigh heavily in the assessment. It is unclear whether RAE occurs around the age of applying for flight school for the type of test that are in use. There is no such thing as a gifted educational program for operational tests. Still, the above-mentioned maturational hypothesis may via the development of other cognitive abilities (or general intelligence) make a difference.

The present paper makes a distinct contribution to the understanding of the RAE. Understanding the RAE in emerging adulthood is crucial, especially for operational abilities (not represented in traditional higher education) and cognitive abilities. Crucially, in the present sample, most candidates are emerging adults between 18 and 25, and a possible fading of the RAE with increasing chronological age may be observable. Specific outcome variables of interest are multitasking score, spatial ability– which have not been explored from an RAE perspective to our knowledge- and different [conceptually distinct] cognitive abilities as well as likelihood of getting accepted for higher education in commercial aviation. The analyses will allow for distinction between these constructs.

## 2 Materials and methods

The test battery in focus in the present study is used in the selection of ab initio commercial pilots applying for pilot training at Higher Vocational Education (HVE; in Swedish: Yrkeshögskolan), a post-secondary form of education that combines theoretical and practical studies in close cooperation with employers and industry (programs are offered in specific fields where there is an explicit demand for competence).

### 2.1 Participants and procedure

The data in the present study was collected between January 1, 2009 and December 31, 2019. It comes from 1113 candidates aged between 18 and 48. The candidates applied for HVE in commercial aviation at Lund University School of Aviation (LUSA). LUSA has provided HVE since 2009. LUSA uses a stepwise elimination process in collaboration with Scandinavian Institute of Aviation Psychology (SIAP).

Candidate consent was waived by the ethics committee due to all individuals being unable to be retraced in the project. The only data used in the present study was test related and date of birth, no other individual data was collected. Results are presented only on an aggregated level without the possibility of identifying the results of specific individuals. The research project that the present study is part of concerns the validity of aptitude tests, including their structure and content; not individual results. The presentation of the results and the data handling make it impossible to identify individuals, and the privacy of research subjects is well protected.

### 2.2 Test Battery

Firstly, candidates perform Written Tests of different cognitive abilities (described below). After that the successful candidates perform a Multitasking test and a joystick test of manual spatial ability (both described below). A combination of written cognitive tests are used and have been found to be predictive of training performance in both commercial aviation [27] and air force selection [28].

#### 2.2.1 Written Tests

The different written tests were designed with the intention of capturing distinct subfactors/abilities.

• *Perceptual speed.* Find letter next to number in figure on busy page, write down the letter.
• *Memory retention.* Read a text about a flight, perform a 2-min distractor task, respond to multiple choice questions about text.
• *Spatial ability* (mental rotation). Folding a 3D figure mentally in accordance with a 2D pattern and choose between four alternatives as to which paper corresponds to figure.
• *Logical ability/Matrices* (abstract problem-solving ability). Identify next in sequence of abstract images in an incomplete matrix.

#### 2.2.2 Multitasking test

The multitasking test was designed to capture certain operational abilities such as multitasking ability, perceptual speed, and the ability to divide attention. The test was performed on a Personal Computer. The test duration was 330 s and the candidates performed five tasks simultaneously. The tasks were: (i) to plot coordinates on a grid when a red lamp switched to green, (ii) to write down a number appearing in an arrow that moved up and down, (iii) to write down answers to mental arithmetic [29]questions on a sheet of paper without prompt from the computer (iv), to state the position of an indicator verbally and point towards an up/down button on the screen whenever an indictor moved above or beneath a designated area, (v) to answer questions which appeared on the screen verbally (e.g., *What is the capital of Germany? What day was it two days ago? 13 times 11?*). The candidates were instructed to work with the different tasks to the same extent and to not neglect anything. The different tasks were intentionally set at different levels of auditory and visual signal strength. Part of the challenge for the candidates was to adequately prioritize tasks that did not automatically draw their attention. The difficulty of the individual tasks was set at a [lower] level where maximum problem-solving ability should not matter.

#### 2.2.3 Joystick test of manual spatial ability

Target (The joystick test) was designed to capture spatial and manual ability in and of itself and under simultaneous increasing taxing of the working memory. The test was performed on a Personal Computer. The test duration was 300 s, and the candidates were instructed to simultaneously answer questions they were asked verbally (e.g., *Spell Stockholm backwards! What is 13 times 11? Repeat the following numbers backwards: 9381).* The candidates scored points through landing on a red or green target (which shifted during the test) by using a joystick while avoiding crashing into three moving obstacles.

### 2.3 Data analysis

Relative age was coded on a scale from 1 (born in December) to 12 (born in January), meaning that a higher value indicated higher relative age. Correlations with performance scores (the four written tests, multitasking, and target, all performance scores were standardized) were estimated with Pearson's correlation coefficient. An aggregated performance score was estimated through factor analysis employing the minres factoring method and the regression method for estimating factor scores, available in the fa-function in the psych package [30] The effect of relative age (as a predictor) on odds to be accepted to LUSA (dichotomous outcome variable) or not was estimated with logistic regression. Descriptive statistics between study variables were calculated and chronological age was also tested as a predictor for all outcome variables. Analyses and illustrations were conducted with R 4.3.1 statistical software [31].

## 3 Results

**Fig. 1:**
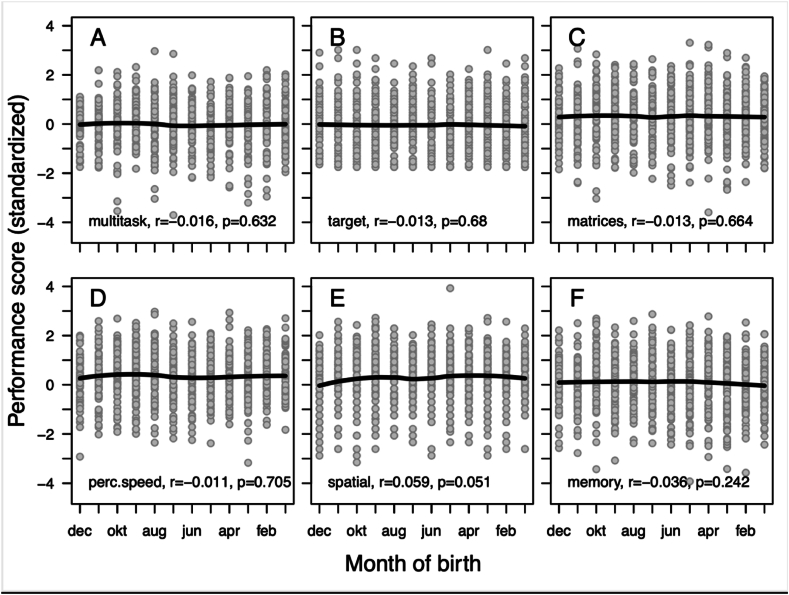
Associations between relative age (Month of birth) and standardized scores for (A) multitasking, (B) manual spatial ability (target), (C) Logical ability (matrices), (D) perceptual speed, (E) spatial ability (spatial), and (F) memory retention (memory).

Of the 1113 candidates with data on relative age (i.e., month of birth), 949 were male (85.3 %, mean age = 23.5 years, range = 17–50 years) and 164 were female (14.7 %, mean age = 21.8 years, range = 18–39 years). Descriptive statistics for and correlations between study variables are presented in Table 1. Chronological age had an association (positive) only with the spatial performance score. Relative age had no statistically significant correlation with any of the six performance scores, which is further illustrated in Fig. 1. Neither did relative age have an association with an aggregated factor score of the six performance scores (*r* = −0.009, *p* = 0.791). Furthermore, relative age could not predict if the candidates were accepted to LUSA or not (*b* = 0.036, *p* = 0.210).

**Table 1:** Descriptive statistics for and correlations between study variables.

Variable	M (SD)	2.	3.	4.	5.	6.	7.	8.	9.
**1. Male**	0.85a (0.36)	0.11^a^	0.06^a^	0.08^a^	0.41^a^	0.04^a^	−0.07^a^	0.11^a^	0.05^a^
**2. Age**	23.24 (5.06)		0.01	0.04	−0.01	−0.02	0.01	0.09^a^	0.05
**3. Rel. age**	6.88 (3.36)			−0.02	−0.01	−0.01	−0.01	0.06	−0.04
**4. Multitask**	0.00 (1.00)				0.27^a^	0.37^a^	0.44^a^	0.26^a^	0.35^a^
**5. Target**	0.00 (1.00)					0.25^a^	0.13^a^	0.31^a^	0.21^a^
**6. Matrices**	0.00 (1.00)						0.45^a^	0.50^a^	0.31^a^
**7. Perc. speed**	0.00 (1.00)							0.39^a^	0.31^a^
**8. Spatial**	0.00 (1.00)								0.26^a^
**9. Memory**	0.00 (1.00)								

a Percent male.

a p < 0.05.

## 4 Discussion

The aim of the present study was to scrutinize the tests in use in commercial aviation and explore whether there is a bias based on birthdate and how it might impact outcomes and by extension flight safety. No such bias was identified. Specifically, there was no RAE for neither any of the six performance scores, nor for the aggregated factor score of the six performance scores, nor for admission into flight school. These findings demonstrate the context dependency of the RAE identified in other samples and reveal limitations of the mechanisms which have previously been put forward. With a sample size of over 1100 aspiring pilots and the rigorous test procedures in use in aviation, the present study makes a distinct contribution on solid ground. The ambitious data collection performed for the present study allows for a unique contribution to the field. Operational tests are in use in numerous high-risk contexts (e.g., aviation, nuclear energy, military) and are vastly different from aptitude tests of grades in traditional higher education. Bluntly put, the participants in the present study are cognitively and operationally strong and the test scores captured (for most participants in emerging adulthood) are most likely not results of [exposure to] the educational system. No effect of maturation, neither through advantageous exposure in an age group nor through chronological ageing was identified. This is a positive finding with respect to potential bias in the test batteries that are devised to identify potential.

So, what are the results indications of? The results may be an indication of the RAE having faded in early adulthood. Previously, findings on university students have also identified a fading of the RAE, with the authors concluding that some intellectual qualities peak between 18 and 21 and then decline [32]. However, another way of interpreting that finding is simply that the effect of general intelligence becomes more and more predictive of outcomes across the lifespan. People tend to gravitate towards their intelligence level in terms of achievement [33]. That does not necessarily mean that intellectual abilities peak at early adulthood or that the same goes for all abilities. However, as the test battery in the present study assesses abilities that are not designed to predominantly be a consequence of education, rather an indication of “raw” ability, the absence of significant differences points away from and RAE in “raw” abilities. At least to a larger extent than in abilities dependent on exposure (e.g., time in education, selected groups in education; ice-hockey talent groups). Interestingly, education may not enhance intelligence in a way previously assumed [34], which means that the maturational hypothesis may be less important for RAE than if that were the case. Educational level is closely linked to intelligence (e.g., Dreher & Bretz, 1991), which means that people may gravitate towards a matching achievement level– as mentioned above– regardless of birth month, at least over time.

However, RAE can potentially persist if there is competition between candidates in selection followed by relevant exposure as hinted to above (e.g., training/schooling). For instance, selected ice-hockey players keep playing and in developmental contexts and advantages accumulate, as suggested by Merton (1968). The present study's findings do not point towards that kind of unfair cumulative advantage starting at selection in operational contexts. Thus, one way of seeing RAE is that it is a selection variable. Relative age matters at the time of selection in many instances, the findings in the present study show that for operational testing and in young adults it does not matter. One reason that the maturation hypothesis has been put forward to explain RAE is that skill attainment at one stage raises skill attainment at later stages of the life cycle [35]. However, in careers more demanding of a general level of intelligence rather than specific skills development happens sooner or later anyway, also for people born later in the year. In relation to Gladwell's [1] point it is also worth noting that it is considerably harder (i.e., statistically less likely) to become a professional ice-hockey player than commercial pilot. In other words, the relative age effect may play a stronger role high up on the bell-curve of whatever ability it portrays. Similarly, the RAE has been observed in Nobel prize winners [36], obviously an extremely narrow selected sample high up the bell-curve.

As mentioned above, a study framed in response to parents enrolling a child into kindergarten at a later stage, found no association between RAE and completion of a research doctorate [26].Yet, that level of achievement may be beside the point. Most people who achieve that level of attainment may have cognitive– and other abilities– to spare. That is, relative late-bloomers (in a high-intelligence group) are not excluded from education since they are competitive enough despite being relatively younger.

The very best may even benefit more from more stimulation early on in life, smart relatively younger students (prodigies) may develop even faster. One finding from a Dutch sample showed that for adolescents who repeated a grade, an inverse RAE was found on achievement and reduction in depressive symptoms [37]. One interpretation is that it is not ideal to be too old in comparison to one's peers. To have repeated a grade can be indicative of other risk factors with respect to attainment. Evidently, the RAE does not exist in a vacuum. The RAE has been shown to depended on socioeconomic status and type of institution on academic performance [38]. Another angle to interpret the results is that they.

Fit with a large body of evidence demonstrating the tendency of intelligence, a very genetic trait, to overpower other factors as people age. Intelligence has been found to be 20 % genetic in infancy to 80 % later in life and to be associated with education and social class [39]. It is possible that the findings of the present study are influenced by the Matthew Effect, for instance through socio-economic position. However, experience-embodied variables contributing to the Matthew Effect, which have been found to matter in education [20] do not seem to matter for operational and cognitive abilities in young adults in Sweden, in terms of relative age (if being relatively older was associated with maturation and in turn receiving more attention and privilege in education, generating a cumulative advantage).

Gladwell's [1] main point; the fact that individual achievement happens in an ecology of others' activities, is important. Although RAE has been found to weaken over time [21], the effect of being included or excluded from contexts where ideal development occurs may have life-long consequences. Exclusion in education or sport should not be trivialized. It is probable that younger students develop negative behavior and attitudes towards education and self-competence, because of their experiences from education [7].

The findings in the present study may be indicative of less barriers than what was feared at one point for relatively younger students. At least in terms of abilities. The fact that chronological age was neither correlated with aggregate score nor with admission is further indication that it is not a question of maturation. Neither relative age nor chronological age predicted performance in any meaningful way. That is good news for a test battery designed to identify individuals with potential that are going to have long and safe careers in aviation.

For the RAE to persist, competition between candidates in selection and subsequent relevant exposure (e.g., training/schooling), may be required. Overall cognitive ability and other factors are likely more predictive of success when the relative size of enhancing exposure is not very large. Gladwell's relatively older ice-hockey players played a lot of ice-hockey in advantageous contexts. There is no guessing what month the pilot is born.

## Data availability statement

Data will be made available on request.

## Funding

This research was funded by Trafikverket, grant number (TRV) 2020/25551.

## Ethics declarations

This study was reviewed and approved by the Ethics Committee of Karolinska Institute with the approval number DNR: 2022–02047.

## Informed consent statement

Informed consent was not required for this study because the only data used in the present study was test related and date of birth, no other individual data was collected. Thus, candidate consent was waived by the Ethics Committee. Data was anonymized prior to being analyzed. Names were replaced with candidate numbers.

## CRediT authorship contribution statement

**Emil Lager:** Writing – review & editing, Writing – original draft, Methodology, Funding acquisition, Conceptualization. **Kimmo Sorjonen:** Writing – review & editing, Methodology, Formal analysis, Conceptualization. **Marika Melin:** Writing – review & editing, Supervision, Project administration, Methodology, Funding acquisition, Conceptualization.

## Declaration of competing interest

The authors declare that they have no known competing financial interests or personal relationships that could have appeared to influence the work reported in this paper.
